# 396. Blastomycosis in Vermont and New Hampshire

**DOI:** 10.1093/ofid/ofaf695.134

**Published:** 2026-01-11

**Authors:** Albert Wu, Lindsay Smith, Jessica Crothers, Rebecca Wang, Isabella Martin

**Affiliations:** University of Vermont, Burlington, Vermont; University of Vermont Medical Center, Burlington, VT; University of Vermont Health Network, Burlington, Vermont; Dartmouth-Hitchcock Medical Center, Lebanon, New Hampshire; Dartmouth Hitchock Medical Center, Lebanon, New Hampshire

## Abstract

**Background:**

*Blastomyces* spp. is endemic in northern Vermont. Diagnosis of blastomycosis can be challenging given its variable clinical presentation. Scant data exist regarding the demographics, diagnostic methods, clinical presentation, and treatment for blastomycosis in northern New England.

**Figure:**
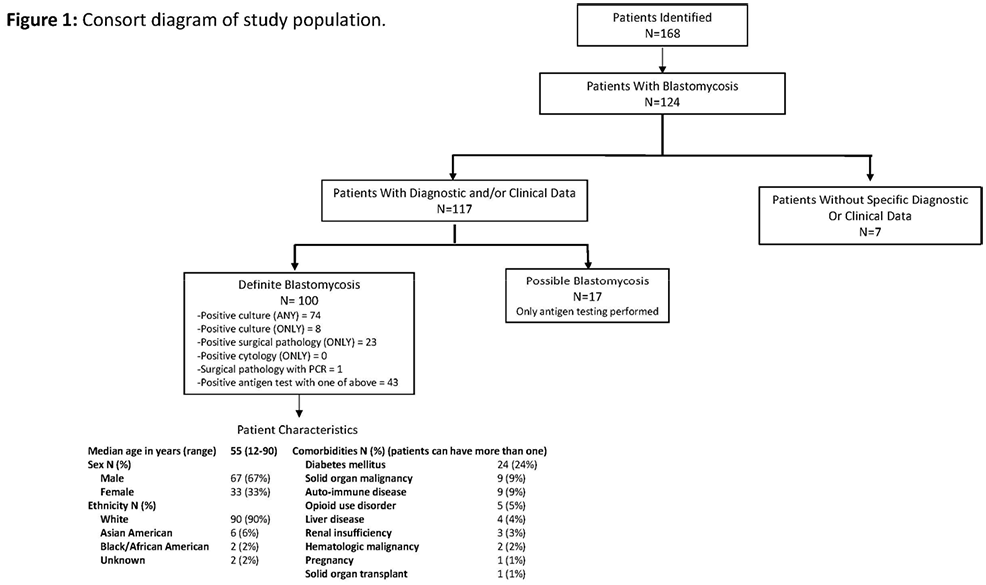

**Figure 2:**
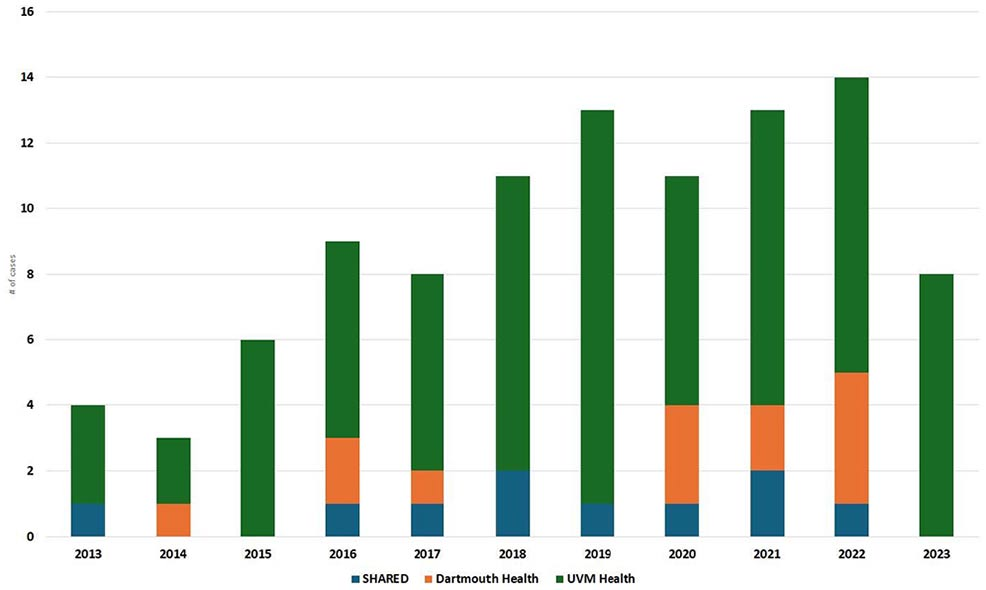
The Number of Definitive Cases of Blastomycosis by Year

**Methods:**

In this descriptive, observational, retrospective study, cases of blastomycosis from 2013 – 2023 were identified through review of electronic medical records at Dartmouth Hitchcock Medical Center and University of Vermont Medical Center. Patient demographic, clinical, diagnostic, and treatment data were collected. Inclusion criteria for case-defined blastomycosis required growth in culture, and/or identification of morphologically consistent yeast forms in surgical pathology or cytology specimens from patients with available clinical data. Data were analyzed with Excel v2018. Ethical approval was obtained by both universities’ institutional review boards.

**Figure:**
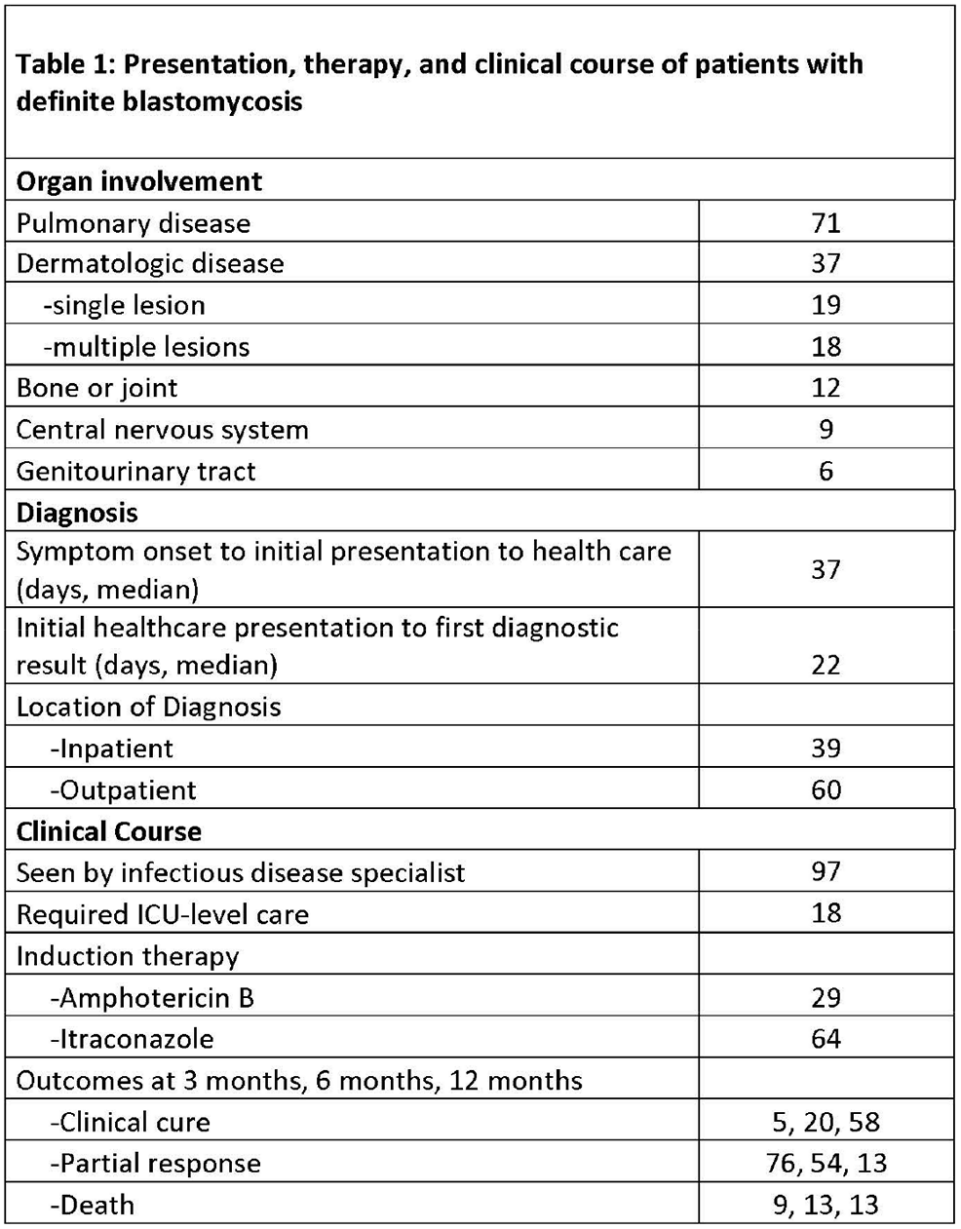

**Figure:**
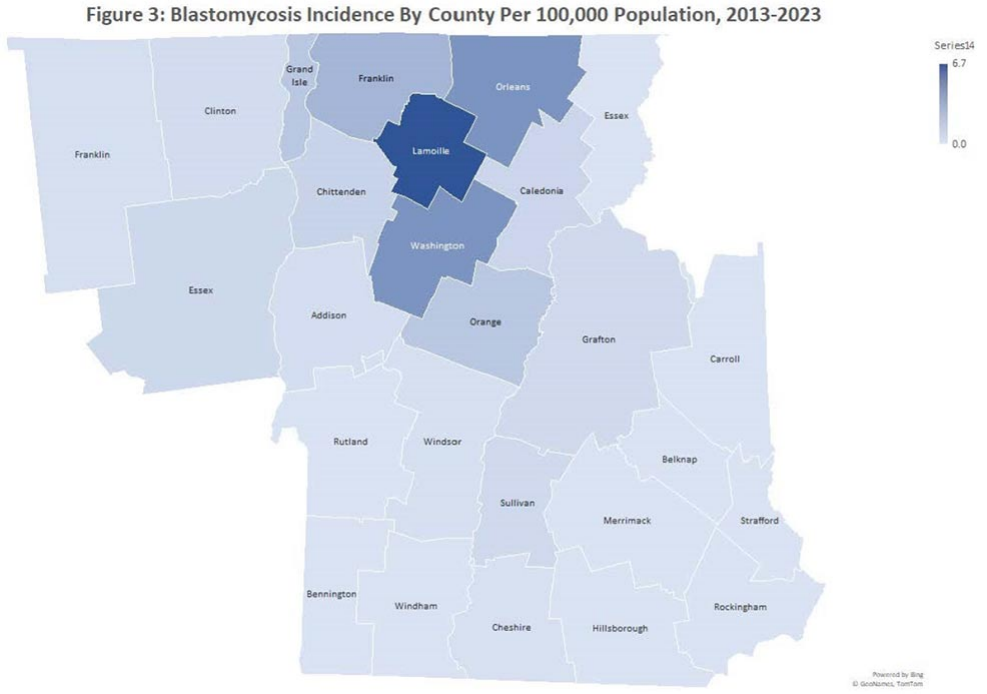

**Results:**

100 patients were identified with blastomycosis, with 22 identified solely by surgical pathology, 8 solely by culture, and the remainder by combination culture and pathology/cytology (Figure 1). There was a sustained increase in case counts after 2015 (Figure 2), with highest incidence in central Vermont, Lamoille County (Figure 3). Median age was 66 (range 12-90), with 67% male and 90% white. Systemic (41%), pulmonary (38%), and dermatologic (42%) symptoms predominated, while 8 patients were asymptomatic. 71 patients had pulmonary blastomycosis. Median time from symptom onset to presentation was 37 days, with an additional median time to diagnosis of 22 days. Initial therapy included liposomal amphotericin B (29%) and itraconazole (64%) (Table 1). 13 patients, 12 of whom had pulmonary disease, died, all within 6 months of diagnosis. At 12 months, 58 patients achieved clinical cure, 13 had partial response, and the rest were lost to follow-up.

**Conclusion:**

The incidence of blastomycosis was variable by county and highest in central Vermont, with pulmonary and dermatologic manifestations being most common. Multiple modalities were used to diagnose blastomycosis. Despite prolonged time to diagnosis and treatment, 12-month survival rate in this cohort was > 85%.

**Disclosures:**

All Authors: No reported disclosures

